# Preparing Students for a More Public Health–Aware Market in Response to COVID-19

**DOI:** 10.5888/pcd17.200251

**Published:** 2020-07-09

**Authors:** Kari Fitzmorris Brisolara, Dean G. Smith

**Affiliations:** 1 School of Public Health, Louisiana State University Health Sciences Center–New Orleans, New Orleans, Louisiana

## Abstract

The COVID-19 pandemic has made the public more aware of public health and the role its professionals play in addressing the pandemic. Schools and programs in public health have a new opportunity to recruit, train, and sustain the public health workforce. Academic public health can further educate the public and prepare students for meaningful careers through interprofessional education and practice-based learning.

**Summary**

**What is already known about this topic?**

The COVD-19 pandemic has made many people aware of the importance of the public health perspective to the field of health emergency preparedness as a whole.

**What is added by this report?**

As a result of the pandemic, schools and programs in public health have a new opportunity to recruit, train, and sustain the public health workforce. Interprofessional education and practice-based learning should become an integral part of training, as should recruiting racial/ethnic minority students to address racial/ethnic health disparities in morbidity and mortality.

**What are the implications for public health practice?**

The fields of public health and academic public health should take this opportunity to advance the recruiting, training, and sustaining of the public health workforce.

## Introduction

With the onslaught of coronavirus disease 2019 (COVID-19), the public is now more aware of the field of public health and the role of public health professionals in addressing the pandemic. Public health officials are in the news daily, and news coverage has made the terms “contact tracing,” “disease transmission,” and “flattening the curve” part of our everyday vocabulary. News coverage has also highlighted the relationship between preexisting chronic diseases and the morbidity and mortality of COVID-19 (1,2). The Centers for Disease Control and Prevention (CDC) reported that nearly 90% of COVID-19 hospitalized patients had one or more chronic conditions, nearly half had hypertension and/or obesity, and more than one-quarter had diabetes and/or cardiovascular disease (3). With a public more aware of public health, no time has been better to recruit a diverse pool of potential public health students into public health training programs and to provide them with the knowledge, skills, and abilities necessary to be effective practitioners (4). Schools and programs in public health have a window of opportunity to expand interprofessional education (IPE) and practice- and service-based learning to prepare students for meaningful, long-term careers in public health.

## Recruit, Train, Sustain

A decade ago, CDC’s National Center for HIV/AIDS, Viral Hepatitis, STD, and TB Prevention developed a strategic plan for recruiting, training, and sustaining a public health workforce in their unit, a plan that closely matches broad needs across the field of public health (5). Key elements from that strategic plan remain on target. “Recruiting” implies bringing people into public health who might not have previously been aware of the field and the importance of its work. “Training” implies offering the combination of knowledge and skills that enable graduates to make immediate contributions. And “sustaining” implies training that enables graduates to advance in their careers.

COVID-19 is a public health threat for everyone beyond anything we have experienced in decades. People with chronic diseases and the elderly have been particularly affected. Furthermore, this pandemic has highlighted the disparities in our public and personal health systems and the importance of our diversity initiatives. Racial/ethnic minority populations are more likely to live and work in communities with less ability for social distancing and have less reliable access to health services (6). As a result, members of these populations are more likely to be hospitalized when diagnosed with COVID-19 (7,8). In one study, African American patients were less likely to survive hospitalizations for COVID-19, because of older age and a high prevalence of chronic diseases (8). If we are to truly address racial/ethnic health disparities, we must recruit into the public health work force people who represent the communities being served and who are capable of understanding and addressing the needs of their communities (9). Progress has been made in diversifying faculty and students in public health education, yet work remains to establish an academy that reflects the community (10). Schools and programs of public health have clearly stated their commitment to zero tolerance of harassment and discrimination (11). These statements must be transformed into actions for training the next generation of the public health workforce.

With an appropriate pool of students recruited, we turn our attention to training. A key to training is that it should be effective and innovative. To be effective, training should address current areas of greatest need and those projected for the future. Surveys of health departments have consistently indicated the need for leadership positions, epidemiologists, and disease intervention specialists (12). Essential leadership skills include systems, strategic thinking, and change management (13). As we move forward in public health, we need to train leaders who can work across the many sectors that influence the health of populations (14). Beyond the instruction provided to novice students, schools and programs in public health may need to offer more continuing education in advanced skills for active public health professionals. Here, we highlight the need for training through IPE and practice-based learning.

IPE is when students from two or more professions learn about, from, and with each other to enable effective collaboration and improve health outcomes (15). Public health has long been known to play a key role in population health teams. The Interprofessional Education Collaborative (IPEC), in which the Association for Schools and Programs in Public Health was an inaugural member, established competencies that include population health (15,16). The offering of more IPE experiences that highlight population health is one means by which public health can support the transformation of health systems and improve direct patient care (16,17).

The overwhelming effect that the COVID-19 pandemic is having on our health systems, not to mention our personal lives, will raise the visibility of public health in IPE activities in the near term. It will be up to public health practitioners and educators to sustain the visibility of public health in the long term. As with any public health emergency, the current pandemic not only requires a response to medical hazards but also demands collaboration across multiple sectors, the application of public health tools, resilience analysis, and an overall systemization of efforts — all of which would benefit from interprofessional training. An interprofessional team could aid in overcoming common challenges related to any public health emergency, including difficulty with data collection, bias, and incomplete data. Training in rapid needs assessment in current public health curricula is designed to improve understanding of how to prioritize, plan, coordinate, address gaps, avoid duplication, and target populations most vulnerable to a public health emergency. Overall, IPE provides the groundwork in all phases of response, in particular, preparedness. Crucial relationships need to be established before an event to build trust and understand the existing health needs of the community, including identifying people with chronic disease vulnerabilities. In addition, these relationships allow for clear communication among interprofessional team members and between team members and communities, including how best to keep communities informed of new developments. IPE, along with practice and service learning, allows students to develop the necessary skills to be an effective member of the team and determine how to best contribute to planning and response to these emergencies.

In 2014, the Louisiana State University Health Sciences Center (LSUHSC) initiated a large-scale annual IPE event for all first-year students as a way of meeting accreditation standards and achieving IPE learning objectives (18). In the 2019–2020 academic year, we developed a case study on pediatric immunizations based on the 2016 IPEC report (19). The case study presented a new vision for health education and brought public health into a leadership role in discussions among students. The perceptions of the importance of population health and teamwork improved among first-year students (20). By using a population health focus in IPE activities, students learned and applied collaborative practice skills along with recognizing the importance of promoting overall health and well-being instead of just health care. As part of the case study, students considered how the medical, nursing, physician assistant, and public health professions traditionally provide immunizations and related education. Then the questions arose: “What would the impact on health be if more health professionals intentionally participated in the promotion of health through immunization education and support? What if each health professional asked the immunization status of their patients/clients during the medical history review?” Although these questions are certainly being asked in some disciplines and locations, asking these nontraditional questions about the delivery of health care services as part of a national routine might lead to health system transformation. In addition, the evaluation measures of the IPE experience at LSUHSC include not only quantitative metrics on student perceptions of readiness for collaboration and teamwork but also qualitative reflections on their own stereotypes and how they plan to address them. The true measure will be following up after graduation to determine the external career effects of IPE training. Preliminary data from practice-based IPE projects show the potential effect through feedback from a volunteer community member: “It was my pleasure to interact with you over the last few months. In each of the meetings, I found a sincere interesting focusing on my specific health care issues and the means and encouragement to benefit me now and in the future. If the students I met with in this program are any indication of what the public can expect in their future health care providers, then we all will be served better than we are today.”

We define the field of public health by the needs of practice, meaning that practice-based learning is essential to a well-prepared workforce (21). Accredited public health programs require practice in public health through some combination of internships, practicums, fieldwork, practice-based learning, and service learning experiences. There is a role and place for each type of experience, and a responsibility of faculty and practice to know the differences. Internships and related experiences involve immersion in public health, often in an unstructured manner, enabling the student to observe and feel what it means to work in public health. Practice-based learning and service-learning experiences add an explicit academic foundation to a discipline or subject and use practice sites as the laboratory in which skills are honed, work products for public health are prepared (22), and services required for the delivery of a public health program are completed (23).

Early-career public health professionals indicate that experiences in practice are essential to acquiring needed skills and developing mentors and role models (13,24). The presentation of products generated by practice-based courses and the delivery of services though service-learning experiences can also strengthen academic–practice relationships and improve training of the next generation of the public health workforce by establishing connections in the field. These connections further the goals articulated in the late 1990s for merging the “pragmatic needs of the practitioner and the academic quest to advance understanding” (25). These connections also allow for a solid foundation of trust between academic and practice partners, a foundation that can only be built through years of working together toward common goals. By maintaining the ultimate focus of public health on the public and practitioners, academic institutions can better prepare for events such as the current COVID-19 pandemic through the synthesis and dissemination of research findings and training products along with moving new knowledge, particularly as it applies to preparedness and response, from research to practice and policy.

Sustaining the public health workforce is a joint responsibility of academic public health and our state and local health departments and related organizations. Academic public health can recruit and train students through foundational education and opportunities for life-long learning. Advances in public health research can provide practitioners with an expanded set of tools that improve public health outcomes and create on-the-job satisfaction. IPE and practice-based learning provide skills that advance careers, especially in an environment of Public Health 3.0 (14). Public Health 3.0 adds to the core of public health and emphasizes cross-sector collaboration, systems-level action, and other practices (14). Collaboration between public health professionals and professionals in related fields will be essential for sustaining the interest of practitioners in public health and career advancement.

## Implications for Public Health

We echo the call for sustaining the CDC’s Prevention Research Centers and the Health Resources and Services Administration’s Public Health Training Center program (26) and go further to support pandemic preparedness. Shortcomings in pandemic preparedness are attributable to numerous factors, of which a lack of funding for operating public health programs and a lack of funding for training future public health professionals are just two components. We further recognize the importance of making training available to current public health professionals who may lack the full complement of knowledge and skills required to optimally respond to the COVID-19 pandemic and prevent future pandemics. Of course, training is important, but training alone will not resolve all problems. With greater understanding of roles and responsibilities across the health care sector, collaboration efforts would improve. For example, a team with representation across medicine, pharmacy, basic sciences, epidemiology, health care administration, and community health would allow data collection, interpretation, and action to occur seamlessly within the entire scope of the health care community. The expertise of such a team would allow discussions on the status of relief efforts, casualties, availability of essential supplies and personnel, and exposure to physical and psychological stressors.

The COVD-19 pandemic has made many people aware of the importance of the public health perspective to the field of preparedness as a whole. Incorporating the inner workings of emergency preparedness and response into public health academic training will provide students with a stronger foundation and an evidence-based voice. This voice will advocate for creating a role for public health in any emergency planning or response teams at the municipal, state, and federal level. As we have seen with the COVD-19 pandemic, public health training, tools, and skills can provide invaluable perspective on public health emergencies, including how best to prevent them. We can add to the number of trained public health professionals not only by attracting people previously interested in public health but also by recruiting people newly aware of the importance of public health, and we can provide them with the knowledge, skills, and abilities necessary to be effective practitioners.

Finally, we recognize the challenge in the fields of public health practice and academic public health to ensure that resources made available by COVID-19 are used wisely and effectively. We should not waste this time in the spotlight: we should take this opportunity to advance the public health workforce for many years to come. We should be honest with ourselves in assessing the implementation of training programs and be prepared to change directions to optimize the investments of everyone in having a well-prepared workforce (27). Evaluation of how well we do in recruiting, training, and sustaining a public health workforce will be essential, with a focus on how well IPE and practice- and service-based learning contribute to the knowledge and skills of graduates and enable them to sustain effective careers in public health. We can hope and work to ensure that future pandemics are minimized and managed as best as possible.
